# Structure-based design and evaluation of tyrosinase inhibitors targeting both human and mushroom isozymes†

**DOI:** 10.1039/d5md00357a

**Published:** 2025-06-23

**Authors:** Salvatore Mirabile, Giovanna Pitasi, Sonia Floris, Kristina Schira, Lyna Khettabi, Montserrat Soler-Lopez, Jörg Scheuermann, Rosaria Gitto, Maria Paola Germanò, Antonella Fais, Laura De Luca

**Affiliations:** a Department of Chemical, Biological, Pharmaceutical, and Environmental Sciences, University of Messina Viale F. Stagno D'Alcontres 31 I-98166 Messina Italy laura.deluca@unime.it; b Department of Live and Environmental Sciences, University of Cagliari Cittadella Universitaria, SS 554, Km 4.5 09042 Monserrato Italy; c Department of Chemistry and Applied Biosciences, Institute of Pharmaceutical Sciences, ETH Zürich 8093 Zürich Switzerland; d Structural Biology Group, European Synchrotron Radiation Facility 71 Avenue des Martyrs 38000 Grenoble France

## Abstract

Tyrosinase inhibition represents an attractive challenge to fight skin hyperpigmentation for medicinal and cosmeceutical application. We have previously provided insights for the development of novel compounds with a specific shape and functional groups interacting with tyrosinases from distinct sources. We chose to employ the *Agaricus bisporus* tyrosinase (AbTYR) isoform as a cost-effective and rapid screening method prior to carrying out the assay toward human tyrosinase (hTYR). Through this approach, the inhibitor [4-(4-hydroxyphenyl)piperazin-1-yl](2-methoxyphenyl)methanone (MehT-3) has been identified as an effective inhibitor against both TYRs with potency comparable to that of the marketed inhibitor Thiamidol. Continuing our efforts, in this work we designed a focused small series of MehT-3 derivatives that were *in silico* predicted for their ability to occupy the cavity of AbTYR and hTYR; subsequently, we proceeded with the execution of a very simple and efficient synthetic procedure to obtain the designed compounds. As a result, we obtained potent AbTYR and hTYR inhibitors with affinity values ranging from 5.3 to 40.7 μM. Notably, compounds 2 and 3 emerged as the most promising candidates; they exhibited superior activity against hTYR and demonstrated low toxicity as effective antioxidant agents and sunscreen products. Overall, these achievements further strengthened our computational protocol, which could be effectively applied to develop newer tyrosinase inhibitors.

## Abstract 2

Tyrosinase inhibition represents an attractive challenge to fight skin hyperpigmentation for medicinal and cosmeceutical application.

## Graphical abstract

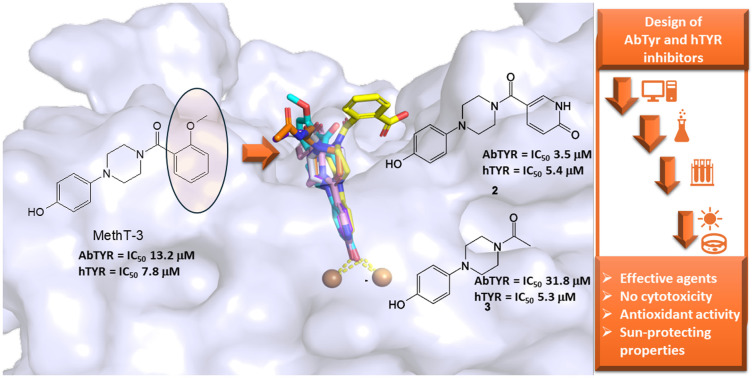

## 1. Introduction

**Fig. 1:**
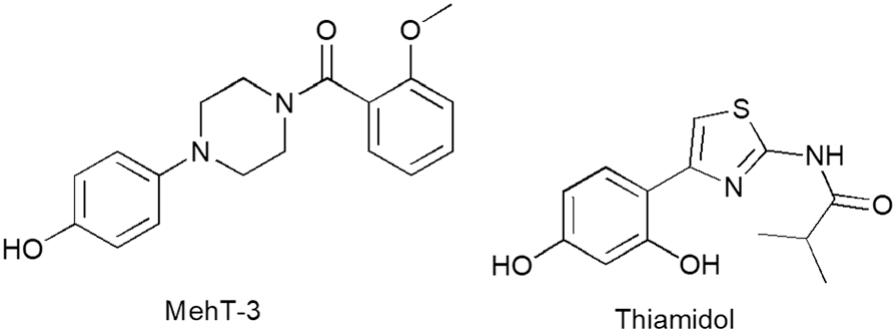
Chemical structure of [4-(4-hydroxyphenyl)piperazin-1-yl](2-methoxyphenyl)methanone (MehT-3) and reference compound isobutylamido thiazolyl resorcinol (Thiamidol).

Metalloenzyme tyrosinases (TYRs, EC 1.14.18.1) are characterized by a catalytic site in which two copper ions are coordinated by six highly conserved histidine residues.^1,2^ TYRs catalyze the oxidation of monophenol/diphenol compounds involved in melanogenesis in many organisms, including vertebrates, bacteria and fungi.^3–5^ In humans, TYR regulates the oxidation of l-tyrosine in the skin pigments eumelanin and pheomelanin, exerting protective effects against ultraviolet irradiation.^6,7^ Therefore, the reduction of hyperactivity of human TYR (hTYR) could provide medicinal and cosmeceutical treatments for skin pathologies related to a high concentration of melanin.^8^ Additionally, the overexpression of hTYR offers a therapeutic opportunity in controlling neurotoxicity that emerges from high concentrations of melanin in neurons.^9^ To date, there are distinct chemotypes of TYR inhibitors (TYRIs) encompassing monophenol/polyphenol-based compounds inspired by natural substrates l-tyrosine and l-DOPA as well as various chemical scaffolds from natural and synthetic sources.^10–12^ Among resorcinol-based compounds isobutylamido thiazolyl resorcinol (Thiamidol®, Eucerin®, CAS 1428450-95-6, Fig. 1) was claimed to possess high activity and physicochemical properties compatible with topical formulations.^13^ Several clinical studies supported the commercial use of Thiamidol in dermocosmetic formulations.^14^ Thiamidol has recently received approval from China's National Medical Products Administration as an active ingredient for skin whitening products, whereas clinical trials are underway in other countries.^13^ As hTYR is difficult to produce on a large scale, the most popular screening assay is generally carried out by using its cheap TYR surrogate from *Agaricus bisporus* (AbTYR, also known as mushroom tyrosinase). To date, AbTYR-based experimental protocols allowed the identification of various TYRIs.^15–20^ However, the strategy of employing AbTYR to identify therapeutics for skin conditions has been widely questioned^21^ as most potent AbTYR inhibitors have failed to effectively target hTYR.^22^ To address this limitation, we have recently published a retrospective computational study highlighting the crucial role of shared residues on hTYR and AbTYR tyrosinase binding affinity; in more detail, we have demonstrated that selected residues (whether conserved or homologous amino acids) are essential targets for enzyme inhibitors that effectively act against both isozymes.^23^ This computational study culminated in the identification of [4-(4-hydroxyphenyl)piperazin-1-yl](2-methoxyphenyl)methanone (MehT-3, Fig. 1), which demonstrated similar inhibitory potency against both AbTYR (IC_50_ = 3.52 μM) and hTYR (IC_50_ = 5.4 μM).^11^ Notably, MehT-3 exhibited biochemical activity on hTYR comparable to that of the marketed compound Thiamidol (IC_50_ = 3.8 μM).^23^ The predicted binding modes of MehT-3 revealed that the 4-hydrophenyliperazine moiety was profitably positioned within the active cavities of both hTYR and AbTYR.^23^ In good agreement with these *in silico* outcomes, kinetic analyses confirmed that MehT-3 affected the catalytic cycle of AbTYR and hTYR, acting as a competitive inhibitor.^23,24^

Herein, MehT-3 compound was considered a good starting point to explore the ability of *in silico* prediction to study affinity on AbTYR/hTYR cavities; in particular, we analyzed the impact of the nature of the amide tail on the pharmacokinetic (PK) properties. After ADMET predictions, molecular docking and molecular dynamic simulations, five analog compounds were synthesized and evaluated using hTYR and AbTYR assays. This approach could provide insights into the reliability of using *in silico* and AbTYR assays as preliminary screening tools before employing the costlier human tyrosinase. Finally, we investigated their effectiveness as sunscreen and antioxidant agents as well as their cytotoxic effects on keratinocyte cell lines.

## 2. Results and discussion

**Fig. 2:**
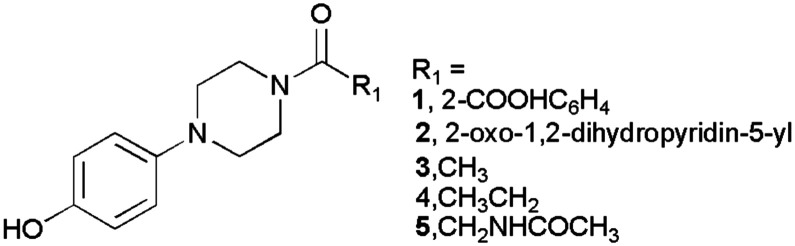
Chemical structure of designed compounds 1–5 inspired by [4-(4-hydroxyphenyl)piperazin-1-yl](2-methoxyphenyl)methanone (MehT-3).

Our goal was to gather more information about chemical structures and molecular sizes capable of inhibiting AbTYR and hTYR. We hypothesized that the poor hydrophilicity of the prototype MehT-3 could compromise favorable recognition within the catalytic pockets of both hTYR and AbTYR, leading us to design five analog compounds bearing different substituents at the N-1 position of the piperazine core (see Fig. 2); consequently, we maintained the 4-hydroxyphenyl ring linked to the opposite N-4 atom as the key feature enabling the interaction with catalytic cavities of both hTYR and AbTYR. We introduced various R^1^ fragments containing either polar groups or shorter carbon chains (1–2 atoms) in place of the 2-methoxyphenyl moiety of MehT-3. We selected readily available reagents from our laboratory that enabled the preparation of new MehT-3 analogs by a simple coupling reaction with the 4-hydrophenylpiperazine starting material (*vide infra* Chemistry section). The selected R^1^ fragments were designed to maintain the crucial structural motifs necessary for creating favorable interaction patterns with hydrophobic and polar residues located at the entrance of hTYR and AbTYR cavities, as detailed in our previous paper for MehT-3.^23^

### 2.1 ADMET properties and drug-likeness prediction

At the beginning of our study, we predicted the PK properties of the designed compounds 1–5. This preliminary *in silico* analysis was aimed at improving the chance to obtain compounds possessing favorable properties suitable for further development as therapeutic agents. We selected several parameters that were calculated using the SwissADME^25^ and pkCSM^26^ web platforms. Specifically, the SwissADME web server (accession date 18 October 2024) predicted that compounds 1–5 were free from PAINS and Brenk structural alerts; moreover, all compounds met the Lipinski's rule of five (more detailed data from SwissADME are reported in the ESI,† Tables S1a and S1b). The PK from the pkCSM web server (accession date 20 October 2024) were compared to those of the two reference compounds Thiamidol and MehT-3 (see Table 1).

**Table 1:** ADMET prediction for compounds 1–5 and reference compounds MehT-3 and Thiamidol

	Absorption		Distribution	Metabolism							Excretion	Toxicity	
	HIA%	Skin permeability (log *K*_p_)	DVDss (human)	CYP2D6 substrate	CYP3A4 substrate	CYP1A2 inhibitor	CYP2C19 inhibitor	CYP2C9 inhibitor	CYP2D6 inhibitor	CYP3A4 inhibitor	Renal OCT2 substrate	AMES toxicity	Skin sensitization
1	90.5	−2.702	−0.459	No	No	No	No	No	No	No	No	No	No
2	92.3	−3.059	0.241	No	No	Yes	No	No	No	No	No	No	No
3	83.5	−3.075	0.105	No	No	No	No	No	No	No	No	No	No
4	92.6	−3.051	0.164	No	No	No	No	No	No	No	No	No	No
5	72.2	−3.790	−0.060	No	No	No	No	No	No	No	No	No	No
**MehT-3**	92.7	−3.303	0.149	No	No	Yes	Yes	No	No	No	Yes	No	No
**Thiamidol**	88.6	−2.899	0.279	No	No	Yes	No	No	No	No	No	No	No

Concerning absorption parameters, all designed compounds were predicted to be absorbable *via* transdermal delivery (see log *K*_p_ values for skin permeability) and they displayed favorable human small intestine absorption, with the exception of derivative 5. All designed compounds possessed VDss values falling in an allowed range except for derivative 1. Additionally, we calculated selected parameters related to the metabolic pathway of compounds 1–5.

The findings suggested that none of the compounds were likely to act as substrates for CYP2D6 or CYP3A4, whereas only derivative 4 was identified as a potential CYP1A2 inhibitor, like Thiamidol and MehT-3.

Regarding the excretion parameters, none of the compounds were predicted to act as substrate of the renal uptake transporter OCT2, thus reducing potential adverse interactions with co-administered OCT2 inhibitors. Finally, all designed compounds 1–5 were not estimated mutagenic substances in the AMES test and none of the compounds revealed skin sensitization as a potential adverse effect. According to these data, compounds 1–5 were predicted to possess acceptable pharmacokinetic profiles.

### 2.2 Computational studies for predicting binding interactions of novel designed compounds

**Fig. 3:**
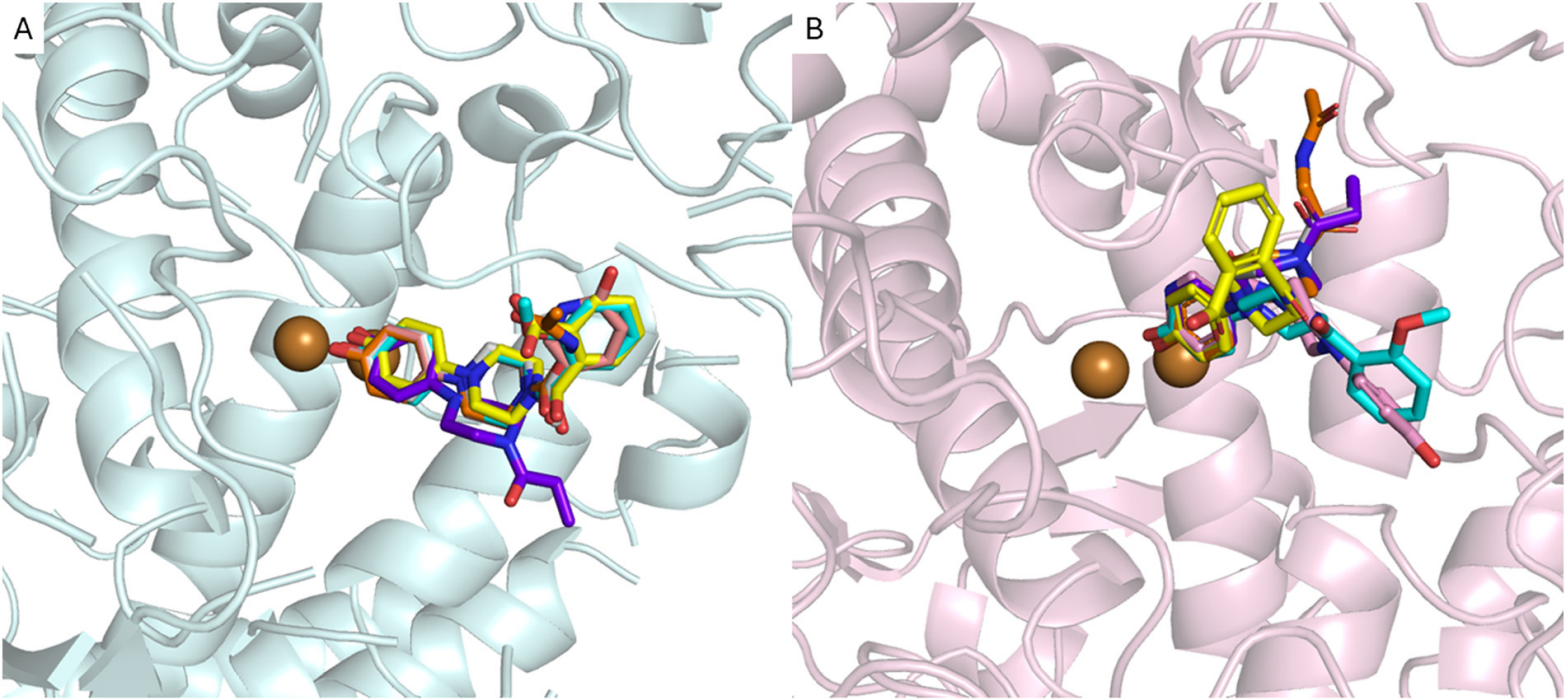
Superimposition of the ligands with MehT-3 (cyan stick) on the AbTYR X-RAY structure, pale-cyan cartoon (PDB 2Y9X) (A) and hTYR homology model, light-pink cartoon (B). The ligands are shown as sticks: 1, yellow; 2, pink; 3, gray; 4, purple; and 5, orange. The copper ions are depicted as brown spheres. The images were created with PyMOL (PyMOL Molecular Graphics System, Version 3.0, Schrödinger, LLC).^30^

In the second phase of this research, we conducted molecular docking and molecular dynamics simulations to preliminarily analyze the interaction pattern of derivatives 1–5 with AbTYR and hTYR isoforms. The computational studies were carried out applying the same protocol that contributed to the discovery of the active compound MehT-3.^23^ In detail, derivatives 1–5 were docked using GOLD v2021 molecular software^27^ into the crystal structure of AbTYR retrieved from the complex with inhibitor tropolone (PDB 2Y9X)^28^ and into the homology structure of hTYR, which we have previously obtained by a multistep computational workflow published in our previous paper^23^ and described briefly in the Experimental procedure section. In depth, docking simulations on the AbTYR cavity revealed that MehT-3 and designed compounds 1–5 established similar ligand–protein interactions, with the common 4-hydroxyphenylpiperazine motif occupying the area of the active site adjacent to a pair of copper ions (Fig. 3A); detailed contacts were also displayed in 2D plots of intermolecular interactions as reported in the ESI† (see Table S2). Only compound 4 oriented its propionyl chain toward a different pocket, but it did not show any steric clashes with the protein, suggesting that it established contacts with AbTYR like the other derivatives, including our active compound MehT-3.

As displayed in Fig. 3B, the docking simulations within hTYR suggested that all five studied compounds were generally found to occupy the catalytic cavity; the tail of the derivatives 1, 3, 4 and 5 adopted a different orientation in the hTYR binding site compared to the parent active compound MehT-3, suggesting that they may interact with the protein in slightly different ways while still maintaining a similar interaction profile with other regions of the active site. Like MehT-3, the new designed compounds 1, 3, 4 and 5 oriented their 4-hydroxyphenyl moieties toward the copper ions (2D plots of intermolecular interactions are reported in the ESI,† Table S3). In contrast, compound 2 assumed a binding mode superimposable with that of MehT-3. Interestingly, compound 2 exhibited the ability to coordinate copper ions through its 4-ketopyridine moiety, whereas the 4-hydropxyphenyl moiety was oriented as found for the 2-methoxyphenyl tail of MehT-3.

**Fig. 4:**
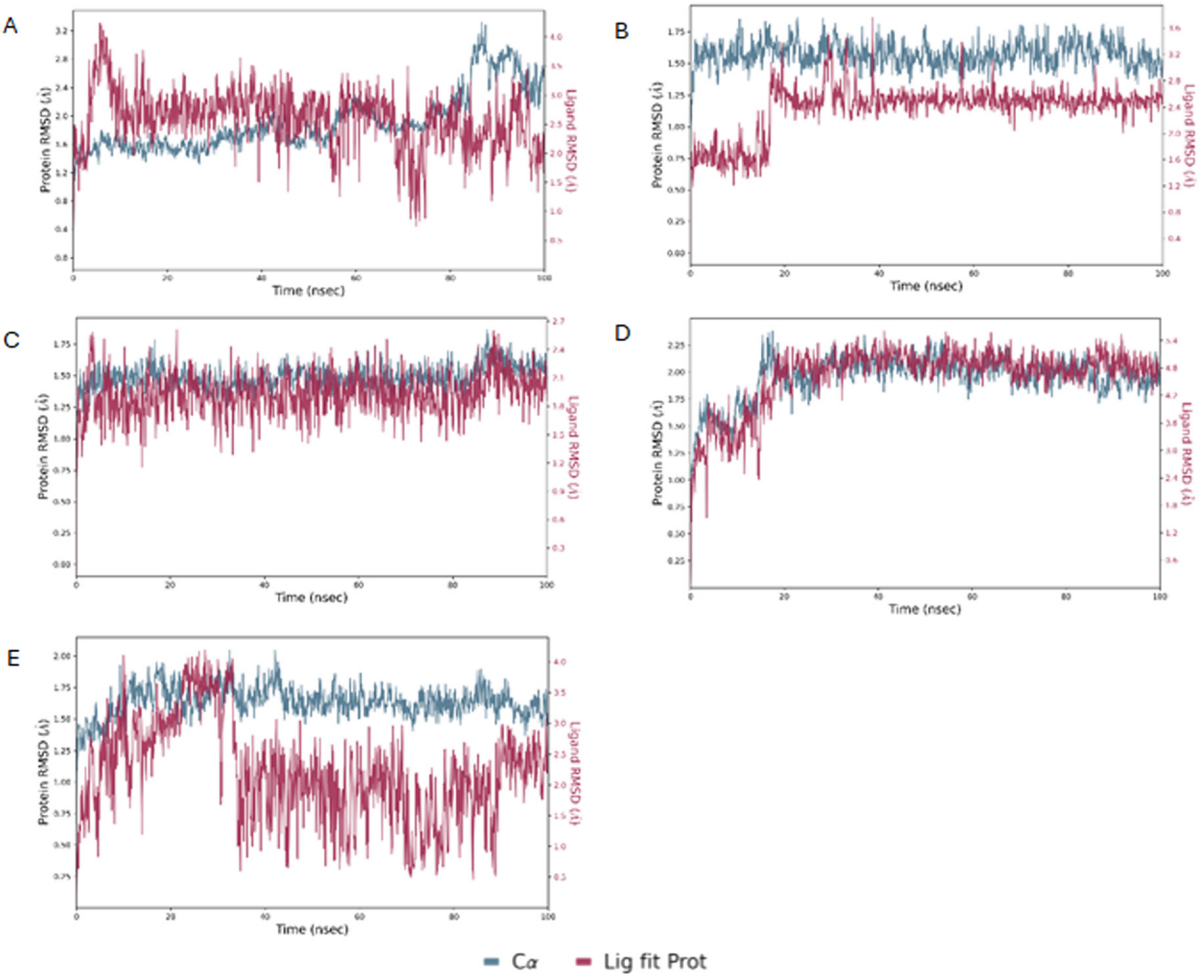
The RMSD of AbTYR protein over a 100 ns MD trajectory for its complexes with derivatives 1 (A), 2 (B), 3 (C), 4 (D) and 5 (E).

To validate the docking results, MD simulations were performed using Desmond software^29^ following the protocol outlined in our previous study.^23^ The MD simulations conducted on AbTYR demonstrated good stability of the protein–ligand complexes, as evidenced by the root-mean-square deviation (RMSD) values shown in Fig. 4. In particular, the protein RMSD did not exceed values of 3.2 Å, indicating that the protein structure remained substantially stable during the simulation, without significant conformational changes. This stability is a positive signal in terms of the reliability of the simulation and the quality of the protein–ligand interaction.

Regarding the ligand RMSD, the values did not exceed 5.4 Å, indicating that the ligands remained relatively stable within the binding site while showing a certain degree of flexibility. These RMSD values for the ligand were adequate, as some movement of the ligand was expected during simulations. Overall, these results suggested that protein–ligand complexes remained stable during simulation.

**Fig. 5:**
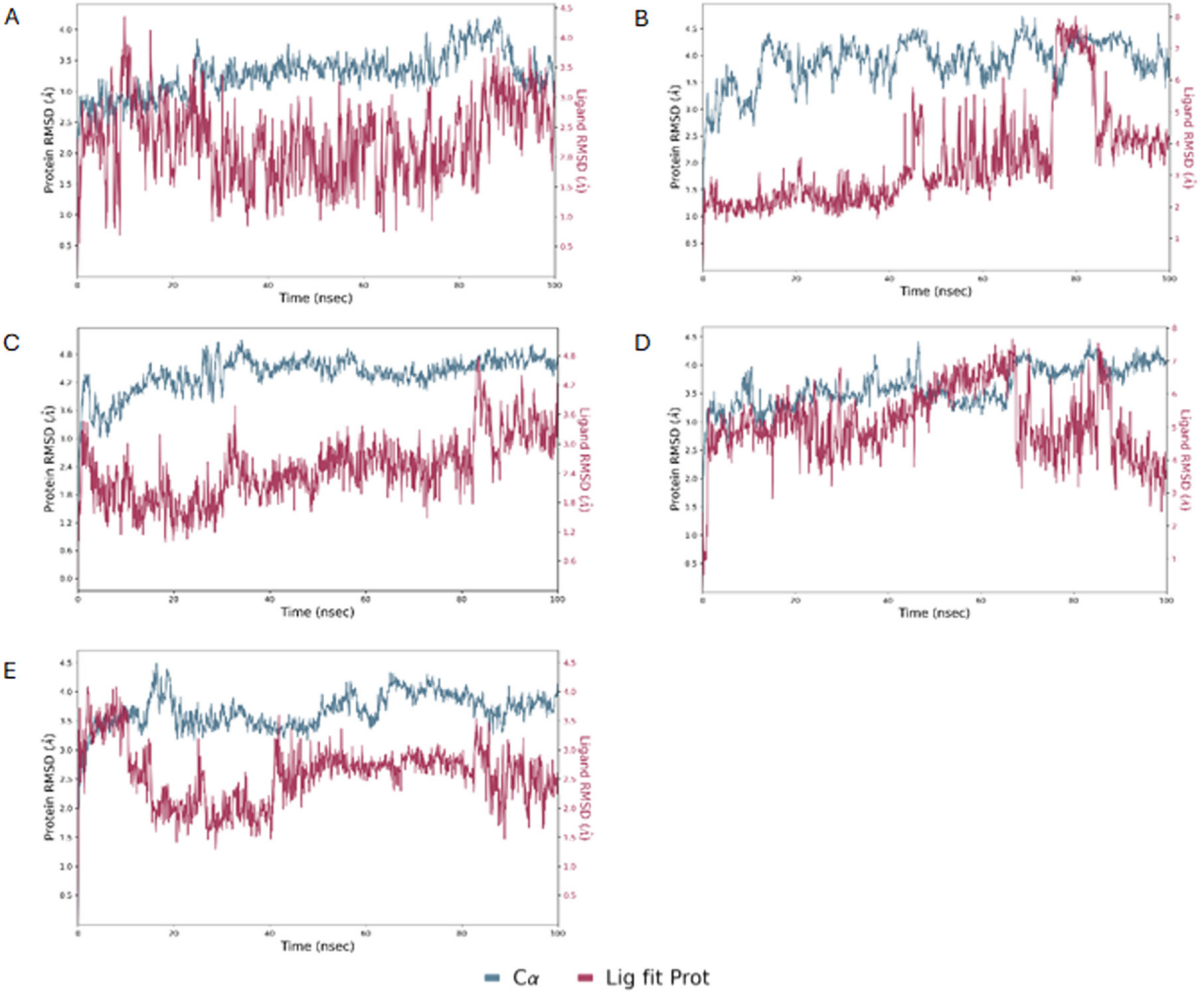
The RMSD of homology modelling of hTYR protein over a 100 ns MD trajectory for its complexes with derivatives 1 (A), 2 (B), 3 (C), 4 (D) and 5 (E).

Molecular dynamics simulations were also conducted on a homology model of hTYR (Fig. 5). The protein RMSD values fluctuated within a range of 1.5–4.5 Å, indicating a relatively stable protein conformation. In contrast, the RMSD values of the ligand exhibited greater fluctuations, especially for compounds 2 and 4. Despite these variations, the ligand RMSD values remained lower than those observed for MehT-3,^23^ indicating improved binding stability. Overall, these computational studies suggested that compounds 1–5 established favorable interactions with both AbTYR and hTYR. Based on these computational studies and considering their favorable ADMET properties and drug-likeness prediction, we were motivated to carry out the synthesis and subsequent biochemical assays for this small series of MehT-3 analog compounds.

### 2.3 Chemistry

**Scheme 1:**
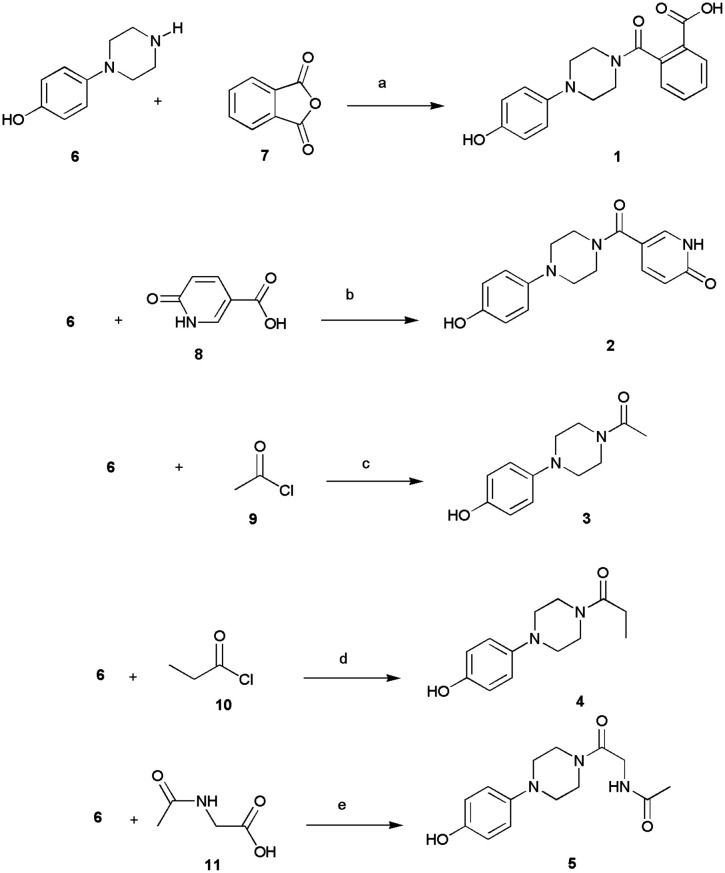
Synthesis of 1–5. Reagents and conditions: (a) DMF, r.t., 15 h; (b) DMF, HBTU, TEA, r.t., 15 h; (c) DMF, r.t., 2 h; (d) THF, r.t., 2 h; (e) DMF, DIPEA, r.t. 2.5 h.

The designed compounds 1–5 bearing different moieties linked to the minimal pharmacophoric requirement 4-hydroxyphenylpiperazine through amide linking groups were synthesized following the synthetic routes summarized in Scheme 1. The amide derivatives 1–5 were prepared by coupling of the 4-(piperazin-1-yl)phenol (6) with suitable reactive fragments 7–11. The desired compounds 1–5 were obtained following distinct reaction conditions based on the most favorable conditions as well as availability of commercial reagents for coupling reactions. The syntheses were conducted at room temperature by using basic conditions and/or the well-known coupling reagent HBTU. In detail, the reaction of amine 6 with phthalic anhydride (7) gave the desired 2-[4-(4-hydroxyphenyl)piperazine-1-carbonyl]benzoic acid (1), whereas the compounds 2–5 were prepared by using carboxyl derivatives 8–11. Finally, we used the starting material acyl chlorides 9–10 to obtain compounds 3–4.

Based on the consideration that the lipophilicity of an active molecule exerts a relevant role in skin penetration as well as in the process of crossing the cell membrane, we chose to experimentally estimate the relative lipophilicity of the synthesized compounds 1–5. We employed reverse-phase thin layer chromatography (RP-TLC); the retardation coefficients (*R*_Fs_) were measured from the chromatograms, thus generating the *R*_M_ values as an estimation of the relative lipophilicity among the homogenous series of the studied compounds 1–5 in comparison with MehT-3. The Experimental procedure (4.3 Lipophilicity) contains a detailed description of the applied methods. The *R*_Ms_ calculation revealed that the studied compounds 1–5 possess *R*_Ms_ values ranging from −0.65 to −0.17; the compounds 2 and 5 possessed lower lipophilicity (−0.60 and −0.65) with respect to the parent compound MehT-3 (−0.17), thus confirming that the introduced moieties generated more hydrophilic compounds.

### 2.4 Inhibition of AbTYR and hTYR by designed compounds 1–5

We assessed the inhibitory effects of compounds 1–5 towards AbTYR and hTYR isoforms, comparing their activity to the reference compound MehT-3 (Table 2).

**Table 2:** Biochemical data of AbTYR and hTYR for compounds 1–5 and Meth-3 as reference compound^23,24^

Entry	AbTYR	hTYR
	IC_50_^a^ (μM)	IC_50_^a^ (μM)
1	40.7 ± 3.19^a^	15.4 ± 1.2^a^
2	13.24 ± 2.24^b^	7.8 ± 0.8^a,b^
3	31.84 ± 3.37^c^	5.3 ± 0.9^b^
4	8.74 ± 0.36^b,d,e^	24.1 ± 3.3^c^
5	4.00 ± 0.25^d,f^	27.2 ± 6.2^c^
MehT-3	3.52 ± 0.25^e,f^	5.4 ± 0.3^b^

a Different letters denote statistically significant differences between compounds within the same column (*p* < 0.05).

Regarding the AbTYR assay, all tested compounds exhibited good inhibitory activity, with a potency ranging from 4.00 to 40.7 μM. The most potent compound 5 demonstrated comparable potency to MehT-3.^24^ Notably, derivatives 2, 4, and 5 exhibited higher inhibitory activity against AbTYR (Table 2)^24^ when compared to kojic acid (KA, IC_50_ = 17.76 μM), which generally serves as the standard reference compound for AbTYR assays. Only compounds 1 and 3 displayed lower inhibitory effects towards AbTYR with respect to MehT-3.

Then, we evaluated the inhibitory potency of all compounds against hTYR using the same protocol applied to MehT-3 in our previous paper.^23^ Notably, as shown in Table 2, all tested compounds effectively inhibited hTYR at low micromolar concentrations, exhibiting IC_50_ values ranging from 5.3 to 27.2 μM. Compounds 2 and 3 exhibited the highest inhibitory activity, displaying a similar potency to MehT-3; furthermore, their efficacy against hTYR was similar to that of the marketed compound Thiamidol (IC_50_ = 3.8 μM).^23^ Taken together, these data confirmed that the 4-hydroxyphenylpiperazine moiety was crucial to exert inhibitory effects towards AbTYR and hTYR; additionally, the replacement of the 2′-methoxyphenyl-substituent of MehT-3 with suitable chemical fragments could enhance the selectivity towards the two isoforms.

### 2.5 *In vitro* determination of antioxidant activity, cell viability, ROS scavenging properties and sun protector factor activity

Considering that melanin synthesis may contribute to oxidative stress and that the redox activities of its intermediates allow the generation of reactive oxygen species (ROS) and/or the reduction of protective antioxidants,^31^ it has been demonstrated that antioxidant agents can help reduce hyperpigmentation and decrease melanin production. Therefore, we conducted a preliminary assessment of the antioxidant activity of all synthesized target compounds by evaluating their ABTS radical scavenging capacity. The ABTS assay is widely recognized as one of the most reliable methods for measuring antioxidants' ability to neutralize the stable ABTS˙^+^ radical cation.

Furthermore, it is well known that radical scavenging ability correlates well with antioxidant activity. The EC_50_ values of antioxidant activity in the ABTS assay are presented in Table 3. All compounds showed good antioxidant effects. Interestingly, compounds 2, 4 and 5 were found to be able to quench the ABTS radical statistically better than the positive control Trolox. Furthermore, compound 3 displayed antioxidant capacity equivalent to that of the reference compound Trolox.

**Table 3:** Antioxidant activity of compounds 1–5

Entry	EC_50_^a^ (μM)
1	17.1 ± 0.7^a^
2	9.5 ± 0.2^b^
3	14.0 ± 0.9^c^
4	10.8 ± 0.3 ^b^
5	10.4 ± 0.1^b^
Trolox^b^	13.0 ± 1.1^c^

a Different letters denote statistically significant differences between compounds within the same column (*p* < 0.01).

b Positive control.^32^

**Fig. 6:**
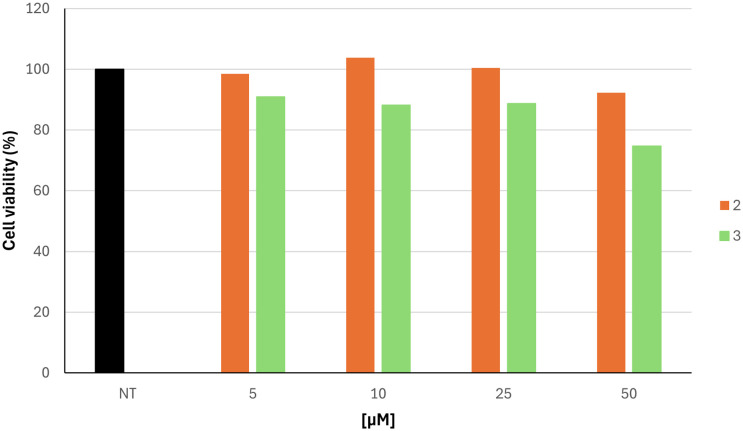
Cell viability of HaCaT cell line after treatment with varying concentrations of compounds 2 and 3, ranging from 0 (NT) to 50 μM.

Based on the inhibitory effects on hTYR and antioxidant properties on the ABTS assay, we focused our attention on compounds 2 and 3, which were further characterized as skin protective agents. In more detail, we evaluated their ability to influence the viability of human skin keratinocyte cell line HaCaT at distinct concentrations ranging from 5 to 50 μM (Fig. 6). Cytotoxicity experiments demonstrated that the two studied compounds were not cytotoxic at concentrations that effectively inhibited hTYR. Cell viability remained above 87% in compound 2 for all concentrations tested. A slight decrease in viability was observed for compound 3 at a concentration of 50 μM, resulting in a percentage viability of 75%.

To further confirm their antioxidant capacity, we carried out an antioxidant assay in the skin keratinocyte cell line HaCaT. The levels of ROS were measured before and after exposure to H_2_O_2_-induced oxidative stress and after treatment with compounds 2 and 3 at various concentrations (5–50 μM).

**Fig. 7:**
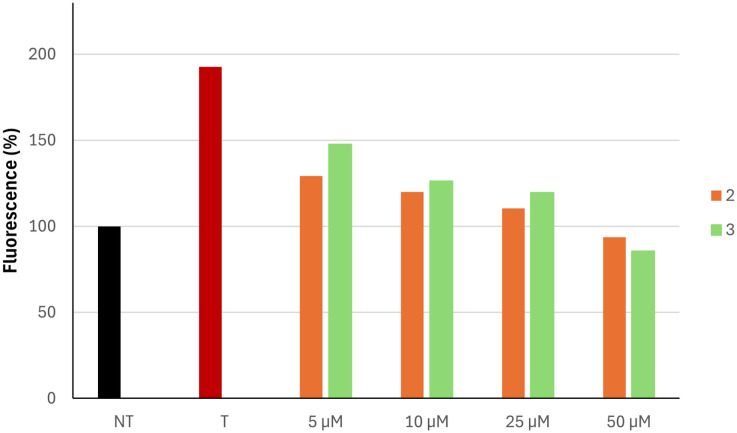
Inhibition of H_2_O_2_-induced ROS generation by compounds 2 and 3 on HaCaT cells. NT, non-treated cells; T, cells treated with H_2_O_2_ only.

As shown in Fig. 7, treatment with the tested compounds 2 and 3 decreased the production of ROS induced by hydrogen peroxide in a dose-dependent manner. These results confirmed the antioxidant activity data obtained *via* spectrophotometric assay (*vide infra*) and suggested that compounds 2 and 3 also reduce the formation of ROS in a cellular system.

Finally, we investigated the sun-protective activity of the compounds by measuring their sun protection factor (SPF) by *in vitro* UV spectrophotometry. The obtained SPF values are reported in Table 4. The sunscreen properties of selected compounds 2 and 3 were evaluated in comparison with those of ferulic acid (FA), caffeic acid (CA) and cinnamic acid (CI), which are commonly used as photoprotective agents in sunscreen formulations.^33^

**Table 4:** SPF values of compounds 2 and 3 and reference compounds ferulic acid (FA), caffeic acid (CA), and cinnamic acid (CI)

Entry	SPF^a^
2	7.36 ± 0.02^a^
3	1.62 ± 0.10^b^
FA^b^	7.5 ± 0.2^a^
CA^b^	6.6 ± 0.4^c^
CI^b^	2.0 ± 0.2^b^

a Different letters denote statistically significant differences between compounds within the same column (*p* < 0.005).

b Data taken from the lietrature.^33^

Compound 2 was found to possess the most effective photoprotective effects, statistically equal to FA. Considering that UV rays are known to contribute to skin aging and oxidative stress, these promising SPF properties might contribute in reducing the absorption of UV rays, so that its antioxidant activity could be indirectly enhanced and consequently skin aging could be reduced.

## 3. Conclusions

In this study, we developed a focused series of five compounds inspired by a previous “hit compound” that had been shown relevant inhibitory effects towards hTYR and AbTYR. The design of new analogue compounds was *in silico* validated by ADMET predictions, docking and dynamic simulations. All synthesized compounds proved to simultaneously inhibit hTYR and AbTYR at low micromolar concentrations. Additional *in vitro* assays revealed that the best active hTYR/AbTYR inhibitors exhibited antioxidant and sun-protecting effects and low cellular toxicity. In conclusion, our findings confirm that the 4-hydroxyphenyl-piperazine moiety offers a nice structural element for further development of therapeutic agents tackling pathologies related to tyrosinase overactivity and that effective inhibition of AbTYR and hTYR requires specific chemical scaffolds with precise structural features and dimensions.

## 4. Experimental procedure

### 4.1 Docking protocol and MD simulation for compounds 1–5 on AbTYR and hTYR

Docking of derivatives 1–5 and their MD studies were carried out following the same protocol used in our previous paper.^23^ In detail, the docking analyses were performed using 100 GA runs per ligand and clustering the poses at threshold values of 0.75 Å. Protein preparation was performed using the Protein Preparation Wizard in Maestro,^34^ applying default settings. All the ligands were prepared using LigPrep^35^ by setting OPLS4 as force field and pH 7.4. For the docking on AbTYR we used the crystal structure of AbTYR in complex with tropolone obtained from the RCSB Protein Data Bank (PDB 2y9X);^28^ its binding site was defined as a 10 Å radius around the coordinates (*x*: 10.021, *y*: −28.823, *z*: −43.596). A modified version of the ASP scoring function was used, including metal coordination terms. This resulted in improved re-docking accuracy of the native ligand, tropolone. For the docking on hTYR we used a homology model prepared and described in detail in our previous paper.^23^ Briefly, six structural models of human tyrosinase were generated from the input file FASTA P14679 downloaded from the UniProt database and using four different software programs, AlphaFold V.2,^36^ SwissModel,^37^ MODELLER through the ModWeb server,^38^ and TopModel.^39^ The results were then aligned using MOE, with SwissModel 1 as the reference (PDB model 5M8L).^40^ The metal centers were standardized by converting Zn^2+^ ions to Cu^2+^ or by sampling Cu^2+^ coordinates from the reference model. Structural preparation involved protonation (Protonate3D), charge assignment (force field: OPLS-AA), minimization, and validation with WHATCHECK^41^ and PROCHECK,^42^ particularly around the active site. The hTYR binding site was defined based on the superposition with l-tyrosine from the BmTYR crystal structure (RCSB code 4P6R),^43^ again using a 10 Å radius. For each analysis, the (best) pose from each ligand was analyzed for protein–ligand interactions using Maestro.^34^

Molecular dynamics simulations were carried out using Desmond 2024 (ref. 29) by setting an orthorhombic box of dimensions 15 Å × 15 Å × 15 Å, OPLS4 as force field and the TIP3P water model. To make the total charge of the system neutral, Na^+^ and Cl^−^ ions were added by maintaining the salt concentration at 0.15 M. The MD simulation was performed for 100 ns using the NPT ensemble class at 310 K temperature and 1.01325 bar pressure. The protocol was reported in a previous work.^23^

### 4.2 Chemistry

Solvents and fine chemicals were purchased from Merck Sigma Aldrich (Milano, Italy) and Thermo Fisher Scientific–Alfa Aesar (Segrate, Italy); both reagents and solvents were employed without purification. Thin layer chromatography (TLC) was carried out using TLC pre-coated silica gel plates (glass sheets) and 20 cm × 20 cm plates coated with C18 silica (HPTLC silica gel RP18) with fluorescence indicator F254 (from Merck, Darmstadt, Germany, Merck, 60, F254). Proton and carbon NMR spectra were recorded on a Varian Gemini 500 instrument (Palo Alto, CA, USA) using DMSO-*d*_6_ as solvent; all spectra were recorded at room temperature. The NMR chemical shifts (*δ*) are expressed in parts per million (ppm) and the coupling constants (*J*) are reported in hertz (Hz). Spectral data are presented in the ESI† (Fig. S1–S10). Melting points were recorded on a Buchi B-545 instrument (BUCHI Labortechnik AG, Flawil, Switzerland) and are uncorrected. The purity of compounds was observed to exceed ≥95% by elemental analyses (C, H, N) recorded with a Carlo Erba 1106 Analyzer.

#### 4.2.1 Synthesis of 2-[4-(4-hydroxyphenyl)piperazine-1-carbonyl]benzoic acid (1)

To a solution of the 4-(1-piperazinyl)phenol (1.4 mmol) in *N*,*N*-dimethylformamide (DMF) phthalic anhydride (1.54 mmol) was added. The reaction mixture was stirred overnight at room temperature. Then, it was quenched with water (5 mL) and extracted with EtOAc (3 × 15 mL). The organic phases were collected and washed with brine (3 × 20 mL); then, the organic phase was dried with anhydrous Na_2_SO_4_. Finally, the solvent was removed *in vacuo*, giving the final product 1 as a pink powder in 45% yield, mp 214–216 °C. ^1^H NMR (500 MHz, DMSO-*d*_6_) *δ* ppm 2.87 (2H, m, CH_2_), 3.02 (2H, m, CH_2_), 3.17 (2H, br s, CH_2_), 3.71 (2H, br s, CH_2_), 6.65 (2H, d, *J* = 8.80 Hz, ArH), 6.78 (2H, d, *J* = 8.80 Hz, ArH), 7.31 (1H, d, *J* = 7.83 Hz, ArH), 7.52 (1H, t, *J* = 7.58 Hz, ArH), 7.64 (1H, t, *J* = 7.34 Hz, ArH), 7.93 (1H, d, *J* = 7.83 Hz, ArH), 8.88 (1H, br s, OH). ^13^C NMR (126 MHz, DMSO-*d*_6_) *δ* ppm 41.1, 46.4, 49.7, 49.8, 115.4, 118.4, 126.8, 128.6, 130.0, 132.4, 138.3, 143.9, 151.3, 166.9, 168.6. Found: C, 66.30; H, 5.52; N, 8.80. Calculated for C_18_H_18_N_2_O_4_: C, 66.25; H, 5.56; N, 8.58%.

#### 4.2.2 Synthesis of 5-[4-(4-hydroxyphenyl)piperazine-1-carbonyl]pyridin-2(1*H*)-one (2)

A mixture of 6-oxo-1,6-dihydropyridine-3-carboxylic acid (1.68 mmol) and the coupling reagent *N*,*N*,*N*′,*N*′-tetramethyl-*O*-(1*H*-benzotriazol-1-yl)uronium hexafluorophosphate (HBTU, 1.68 mmol) in DMF (4 mL) was stirred at room temperature for 1 h. Then, a solution of 4-(1-piperazinyl)phenol (1.68 mmol) and triethylamine (TEA, 2.52 mmol) in DMF (2 mL) was added. The reaction mixture was stirred overnight at room temperature. The mixture was extracted with water and EtOAc (3 × 20 mL). The collected organic layer was washed with brine (3 × 20 mL) and dried with anhydrous Na_2_SO_4_. Finally, the solvent was removed at reduced pressure and the residue was crystallized with Et_2_O, leading to the designed compound 2 as a pink powder in 30% yield, mp 265–268 °C. ^1^H NMR (500 MHz, DMSO-*d*_6_) *δ* ppm 2.94–2.98 (4H, m, 2CH_2_), 3.60–3.63, (4H, m, 2CH_2_), 6.36 (1H, d, *J* = 9.29 Hz, pyridine), 6.64–6.66 (2H, m, ArH), 6.758–6.81 (2H, m, ArH), 7.52 (1H, dd, *J* = 9.54, 2.69 Hz, pyridine), 7.60 (1H, d, *J* = 2.45 Hz, pyridine), 8.92 (1H, s, OH), 11.88 (1H, br s, NHN̲H̲CO). ^13^C NMR (126 MHz, DMSO-*d*_6_) *δ* ppm 46.1, 50.6, 113.4, 115.7, 118.7, 119.5, 137.1, 140.6, 144.1, 151.5, 162.4, 166.4. Found: C, 64.37; H, 5.48; N, 14.21. Calculated for C_16_H_17_N_3_O_3_: C, 64.20; H, 5.72; N, 14.04%.

#### 4.2.3 Synthesis of 1-[4-(4-hydroxyphenyl)piperazin-1-yl]ethan-1-one (3)

To a stirred solution of 4-(1-piperazinyl)phenol (1.68 mmol) in DMF (10 mL) acetyl chloride (1.68 mmol) was added dropwise. The reaction mixture was stirred at room temperature for 2 h and then filtered. The precipitate was solubilized in EtOAc and extracted with a combination of distilled water and saturated NaHCO_3_ solution (2 mL). The collected organic phases were dried over Na_2_SO_4_ and concentrated *in vacuo* to yield the desired product as a pink powder in 38% yield, mp 182–184 °C. ^1^H NMR (500 MHz, DMSO-*d*_6_) *δ* ppm 2.94–2.98 (4H, m, 2CH_2_), 3.60–3.63, (m, 4H, 2CH_2_), 6.36 (1H, d, *J* = 9.29 Hz, pyridine), 6.64–6.66 (2H, m, ArH), 6.758–6.81 (2H, m, ArH), 7.52 (1H, dd, *J* = 9.54, 2.69 Hz, pyridine), 7.60 (1H, d, *J* = 2.45 Hz, pyridine), 8.92 (1H, s, OH), 11.88 (1H, br s, NHN̲H̲CO). ^13^C NMR (126 MHz, DMSO-*d*_6_) *δ* ppm 46.1, 50.6, 113.4, 115.7, 118.7, 119.5, 137.1, 140.6, 144.1, 151.5, 162.4, 166.4. Found: C, 65.33; H, 7.02; N, 12.81. Calculated for C_12_H_16_N_2_O_2_: C, 65.43; H, 7.32; N, 12.72%.

#### 4.2.4 Synthesis of e 1-[4-(4-hydroxyphenyl)piperazin-1-yl]propan-1-one (4)

The suspension of 4-(piperazin-1-yl)phenol (2.5 mmol) in THF (20 mL) and triethylamine (0.5 mL) was stirred for 15 min at 0 °C; then, the solution of propionyl chloride (3 mmol) in THF (20 mL) was added dropwise. The reaction mixture was stirred at room temperature for 2 h. Then, the reaction mixture was concentrated *in vacuo*; Et_2_O was added to the residue giving the crude target product 4 as a pink powder in 75% yield, mp 153–155 °C. ^1^H NMR (500 MHz, DMSO-*d*_6_) *δ* ppm 0.99 (3H, t, *J* = 7.34 Hz, CH_3_), 2.34 (2H, q, *J* = 7.34 Hz, CH_2_), 2.88–2.91 (2H, m, CH_2_), 2.96 (2H, br s, CH_2_), 3.54–3.57 (4H, m, 2CH_2_), 6.67 (2H, d, *J* = 8.80 Hz, ArH), 6.84 (2H, d, *J* = 8.80 Hz, ArH), 8.98 (1H, s, OH). ^13^C NMR (126 MHz, DMSO-*d*_6_) *δ* ppm 9.5 (CH_3_), 25.6 (CH_2_), 41.05, 44.8, 50.6, 51.0, 115.6, 118.7, 143.6, 151.7, 171.5 (C=O). Found: C, 66.39; H, 7.56; N, 11.84. Calculated for C_13_H_18_N_2_O_2_: C, 66.64; H, 7.74; N, 11.96%.

#### 4.2.5 Synthesis of *N*-[2-[4-(4-hydroxyphenyl)piperazin-1-yl]-2-oxoethyl]acetamide (5)

To a solution of *N*-acetylglycine (5.5 mmol) in DMF (5 mL) we added HBTU (5.5 mmol) and DIPEA (0.5 mL); the reaction mixture was stirred at room temperature for 15 min; then, a suspension of 4-(piperazin-1-yl)phenol (5.5 mmol) in DMF (20 mL) was added. The reaction mixture was stirred at room temperature for 2.5 h and detected by TLC. The reaction mixture was filtered, giving the crude product that was purified by treatment with EtOH (5 mL) to furnish the target compound 5 as an off-white powder in 69% yield, mp 240 °C dec. ^1^H NMR (500 MHz, DMSO-*d*_6_) *δ* ppm 1.87 (3H, s, CH_3_), 2.89–2.95 (4H, m, 2CH_2_), 3.55 (4H, d, *J* = 18.59 Hz, 2CH_2_), 3.96 (2H, d, *J* = 5.87 Hz, NHCHC̲H̲_2_), 6.66 (2H, d, *J* = 8.80 Hz, ArH), 6.80 (2H, d, *J* = 9.29 Hz, ArH), 7.97 (1H, br s, NH), 8.87 (1H, s, OH). ^13^C NMR (126 MHz, DMSO-*d*_6_) *δ* ppm 22.6, 40.6, 41.7, 44.3, 50.4, 50.8, 115.8, 118.8, 144.0, 151.6, 167.3, 171.6. Found: C, 60.35; H, 6.72; N, 15.88. Calculated for C_14_H_19_N_3_O_3_: C, 60.63; H, 6.91; N, 15.15%.

### 4.3 Lipophilicity

Experimental relative lipophilicity was determined by employing reversed phase TLC on 20 cm × 20 cm plates coated with C18 silica. The eluant was prepared by mixing acetone and water (2 : 1 (v/v)). The studied compounds and reference substances were dissolved in MeOH to obtain the working solution with a concentration of 2.0 mg mL^−1^. Then, we activated the plates by heating at 105 °C for 1 h. From each working solution, a volume of 0.2 μL was spotted to the plates. The measurements were repeated three times with distinct disposition of the compounds on the plate, so that *R*_F_ values represented the mean values obtained in triplicate. The chromatograms were developed at a distance of 10 cm from the origin in ascending TLC chambers at room temperature. Then, the spots of the substances were visualized by means of a UV lamp at 254 nm. Based on the retardation coefficients (*R*_F_) we calculated the relative lipophilicity *R*_M_ values according to the formula *R*_M_ = log[(1/*R*_F_) − 1];^44^ higher *R*_M_ values predict a higher lipophilicity.

### 4.4 AbTYR and hTYR inhibition

The assessment of inhibitory activity for tested compounds 1–5 towards AbTYR and hTYR was conducted following our previously established protocols.^23,45^ Mushroom tyrosinase (EC 1.14.18.1) was supplied by Merck (Cat. No. T3824). Kojic acid was employed as a positive standard. The intramelanosomal domain of hTYR was expressed and purified as previously reported in the literature.^46^ The reference compound Thiamidol was purchased from BLDpharm (Shanghai, China).

### 4.5 Antioxidant assay

The overall free radical-scavenging capacity of the compounds was evaluated using the ABTS [2,20-azinobis-(3-ethylbenzothiazoline-6-sulfonic acid)] assay according to previously described methods.^45^ The method relies on the capacity of antioxidants to scavenge the ABTS˙^+^ radical. To generate ABTS˙^+^, a 7 mM ABTS solution was mixed with 2.45 mM potassium persulfate in water, and the mixture was incubated in the dark at room temperature for 24 h. Subsequently, 10 μL of sample at varying concentrations was added to 990 μL of the diluted ABTS˙^+^ solution. After 1 min of reaction at room temperature, the absorbance was recorded at 734 nm. The reference antioxidant used was 6-hydroxy-2,5,7,8-tetramethylchromane-2-carboxylic acid (Trolox). Antioxidant activity was quantified as EC_50_, which represents the concentration of compound needed to reduce the initial absorbance by 50%.

### 4.6 Cell viability

The 3-(4,5-dimethylthiazol-2-yl)-2,5-diphenyltetrazolium bromide (MTT) technique on human skin keratinocyte cell line (HaCaT; CLS-Cell Lines Service, Eppelheim, Germany) was employed to assess cell viability as reported by Pintus *et al.*^47^ The cells were exposed to the compounds at concentrations ranging from 0.5 to 50 μM for a duration of 24 h. Afterward, MTT reagent was added to each well, followed by incubation at 37 °C for 3 h. Following incubation, the MTT solution was removed from the culture plate, and 100 μL of DMSO solvent was added to dissolve the water-insoluble formazan crystals formed in the cells. The absorbance was then measured at 570 nm using a microplate reader (VANTAstar_BMG LABTECH GmbH, Offenburg, Germany).

### 4.7 Intracellular ROS levels

Cellular ROS levels were measured using the 2′,7′-dichlorofluorescein diacetate (DCFH-DA) method.^48^ HaCat cells were exposed to different concentrations of the test compounds, and after 24 h, cells were incubated with DCFH-DA (10 μM) at 37 °C for 30 min and then treated with 2 mM H_2_O_2_. The fluorescence intensity of DCF was immediately measured with a fluorescence plate reader (VANTAstar_BMGLABTECH GmbH, Offenburg, Germany).

### 4.8 *In vitro* determination of sun protection factor

The sun protection factor (SPF) of the best compounds was determined using a UV absorbance method according to the methodology described by Mansur *et al.* (1986).^49^ Absorbance measurements were taken in the 290–320 nm range at 5 nm increments with three determinations made at each point.

The SPF was calculated by using the Mansur equation:
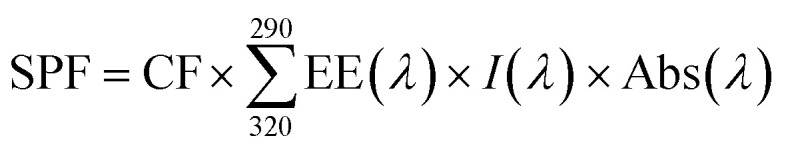
where CF = correction factor (10); EE(*λ*) = erythemogenic effect of radiation with wavelength *λ*; *I*(*λ*) = solar intensity spectrum; and Abs(*λ*) = spectrophotometric absorbance values at wavelength *λ*. The values of EE(*λ*) × *I*(*λ*) are constant, as determined by Sayre *et al.*^50^

### 4.9 Statistical analysis

Statistically significant differences were determined using one-way ANOVA followed by the Tukey multiple comparisons test, performed with Graph Pad INSTAT software v8.2 (GraphPad Software, San Diego, CA, USA).

## Author contributions

SM carried out the synthetic procedure and performed enzymatic assays on AbTYR. GP conducted the computational study. SF and AF performed antioxidant activity, cell viability, ROS scavenging properties and sun protector factor activity. KS performed enzymatic assays on hTYR. LK and MSL provided recombinant hTYR for the enzymatic assays. RG, AF, MPG, JS and LDL wrote the manuscript with input from all authors. All authors have read and approved the final version of the manuscript.

## Conflicts of interest

There are no conflicts to declare.

## Supplementary Material

MD-OLF-D5MD00357A-s001

## Data Availability

The data supporting this article have been included as ESI.†
