# Supplementary material for: Structure-based design and evaluation of tyrosinase inhibitors targeting both human and mushroom isozymes
Source: RSC Med Chem. 2025 Jun 23;16(8):3814–25. doi: 10.1039/d5md00357a (PMC12183826; doi:10.1039/d5md00357a)
Supplement: MD-016-D5MD00357A-s001 [file MD-016-D5MD00357A-s001.pdf]

Supplementary Information (SI) for RSC Medicinal Chemistry.
This journal is © The Royal Society of Chemistry 2025

Supplementary Information (SI).

Salvatore Mirabile, [a] Giovanna Pitasi, [a] Sonia Floris, [b] Kristina Schira,[c] Lyna Khettabi, [d] Montserrat Soler-Lopez, [d] Jörg Scheuermann, [c] Rosaria Gitto, [a] Maria Paola Germanò, [a] Antonella Fais, [b] Laura De Luca[a,*]

[a]*Department of Chemical, Biological, Pharmaceutical, and Environmental Sciences, University of Messina, Viale F. Stagno D'Alcontres 31, I-98166 Messina, Italy*
[b]*Department of Live and Environmental Sciences, University of Cagliari, Cittadella Universitaria, SS 554, Km 4.5, 09042 Monserrato, Italy;*
[c]*Department of Chemistry and Applied Biosciences, Institute of Pharmaceutical Sciences, ETH Zürich, 8093 Zürich, Switzerland*
[d]*Structural Biology Group, European Synchrotron Radiation Facility, 71 Avenue des Martyrs, 38000 Grenoble, France*

Table of contents

Table S1a. Physicochemical properties for compounds 1-5 and the reference compounds Meht-3 and Thiamidol calculated with SwissADME

Table S1b. Druglikeness properties for compounds 1-5 and the reference compounds Meht-3 and Thiamidol calculated with SwissADME

Table S2. 2D plots of molecular docking interactions for AbTYR

Table S3. 2D plots of molecular docking interactions for hTYR

Figures S1-S10. ^{1}H-NMR and ^{13}C-NMR spectra of synthetized compounds 1-5

Table S1a Physicochemical properties for compounds **1-5** and the reference compounds MehT-3 and Thiamidol calculated with SwissADME

	Physicochemical properties					
ID	Molecular weight (g/mol)	Num. heavy atoms	Num. arom. heavy atoms	Num. rotatable bonds	HBA	HBD
**1**	326.35	24	12	4	4	2
**2**	299.32	22	12	3	4	2
**3**	220.27	16	6	2	2	1
**4**	277.32	20	6	5	3	2
**5**	234.29	17	6	3	2	1
MehT-3	312.36	23	12	4	3	1
Thiamidol	278.33	19	11	4	4	3

Table S1b Druglikeness properties for compounds **1-5** and the reference compounds Meht-3 and Thiamidol calculated with SwissADME

	Druglikeness							
ID	Lipinski	Ghose	Veber	Egan	Muegge	Bioavaibility Score	Pains	Brenk
**1**	YES	YES	YES	YES	YES	0.55	0	0
**2**	YES	YES	YES	YES	YES	0.56	0	0
**3**	YES	YES	YES	YES	YES	0.55	0	0
**4**	YES	YES	YES	YES	YES	0.55	0	0
**5**	YES	YES	YES	YES	YES	0.55	0	0
MehT-3	YES	YES	YES	YES	YES	0.55	0	0
Thiamidol	YES	YES	YES	YES	YES	0.55	0	0

Table S2 Schematic 2D plots of the interactions between compounds **1-5** and AbTYR. Pictures were generated by ligand interaction diagram available in Maestro (Schrödinger Release 2024-1: Maestro, Schrödinger, LLC, New York, NY, 2024). Green color represents hydrophobic residues, violet is positively charged residues, red is negatively charged, cyan denotes polar and gray metal ions.

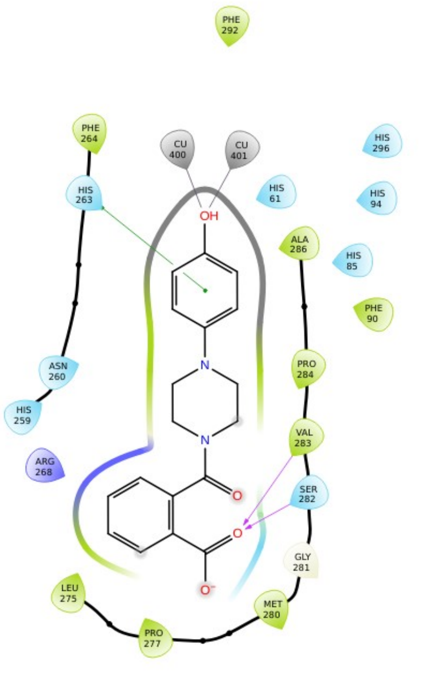

1

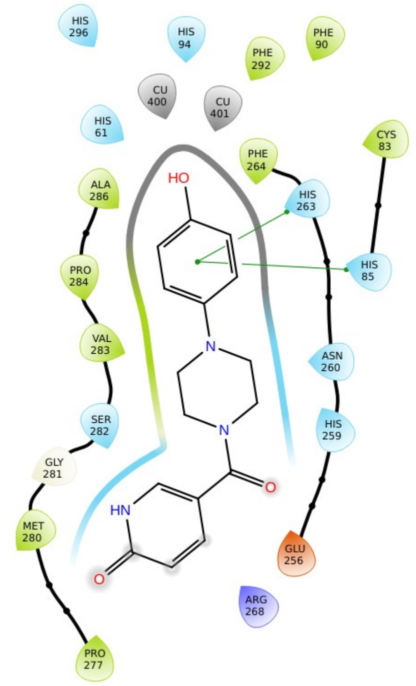

2

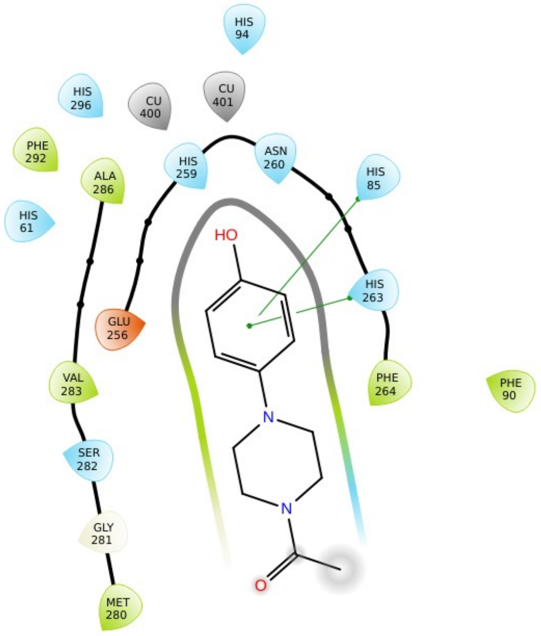

3

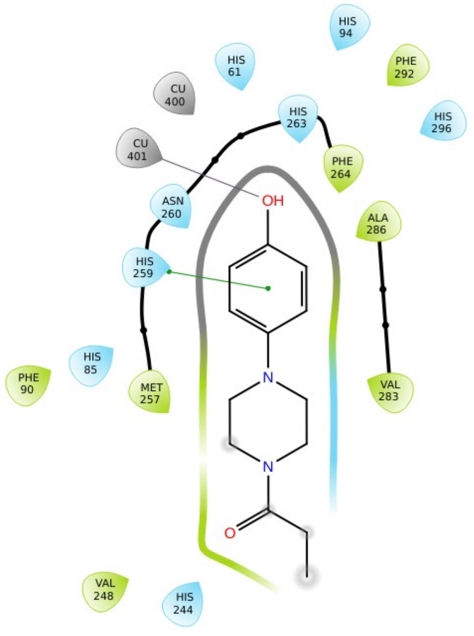

4

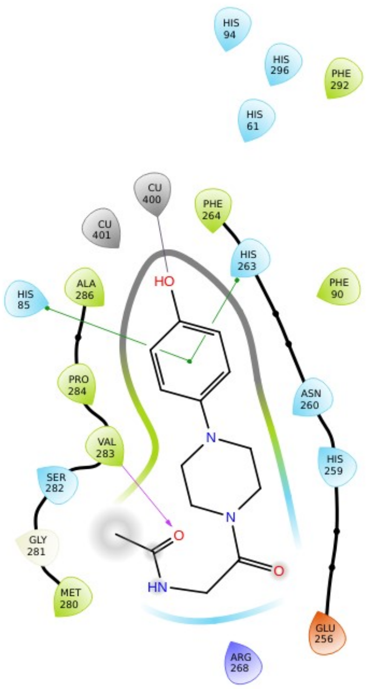
HIS 94
HIS 296
PHE 292
HIS 61
CU 400
CU 401
PHE 264
HIS 263
PHE 90
HIS 85
ALA 286
HO
PRO 284
ASN 260
N
VAL 283
HIS 259
SER 282
N
GLY 281
O
O
HN
MET 280
GLU 256
ARG 268

5

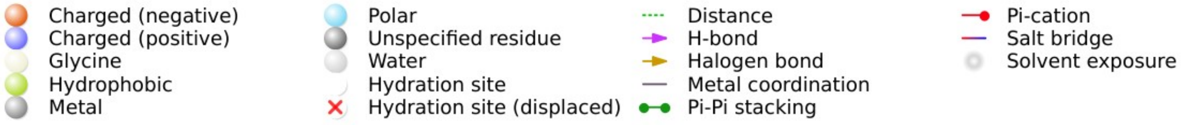
Charged (negative)
Charged (positive)
Glycine
Hydrophobic
Metal
Polar
Unspecified residue
Water
Hydration site
Hydration site (displaced)
Distance
H-bond
Halogen bond
Metal coordination
Pi-Pi stacking
Pi-cation
Salt bridge
Solvent exposure

Table S3 2D plots of the interactions between compounds **1-5** and homology modeling of hTYR. Pictures were generated by ligand interaction diagram available in Maestro (Schrödinger Release 2024-1: Maestro, Schrödinger, LLC, New York, NY, 2024). Green represents hydrophobic residues, violet is positively charged residues, red is negatively charged, cyan denotes polar and gray metal ions.

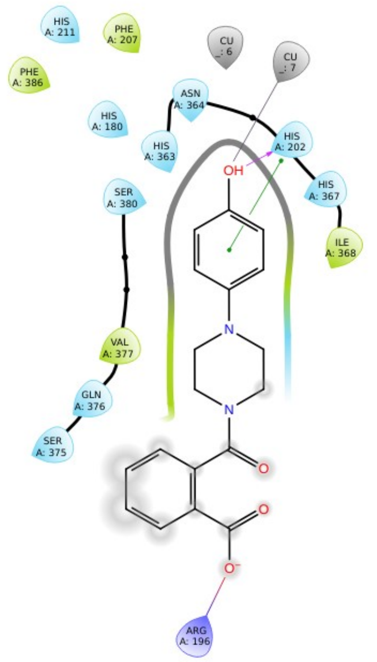

1

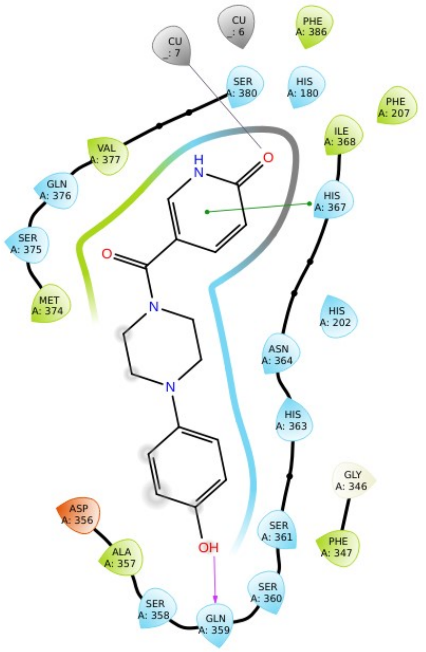

2

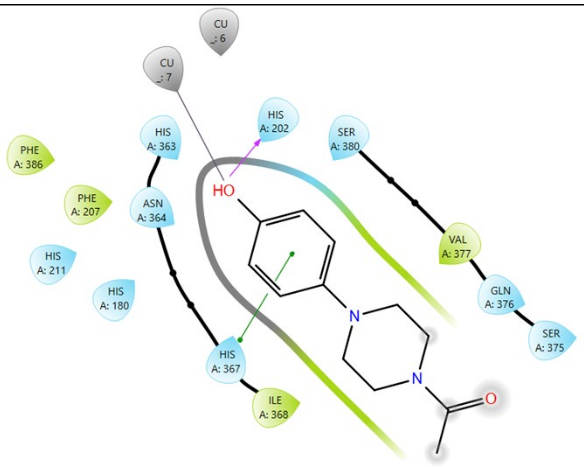

3

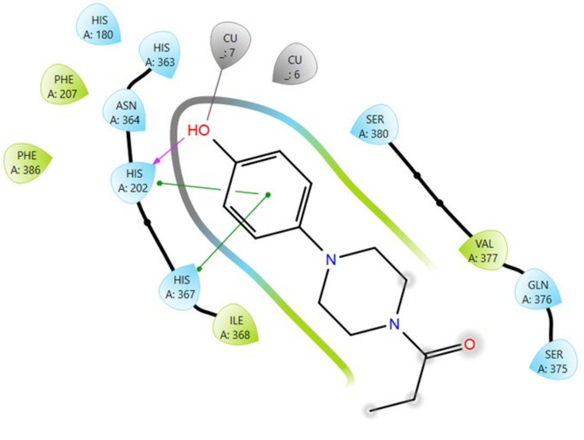

4

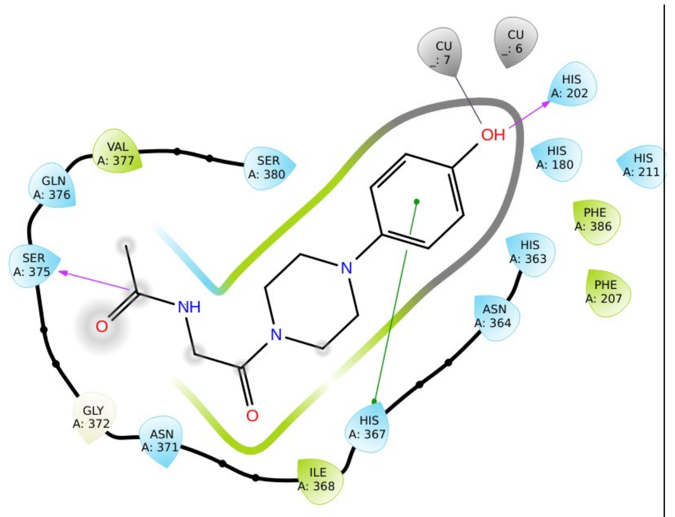
CU
_: 7
CU
_: 6
HIS
A: 202
OH
VAL
A: 377
SER
A: 380
HIS
A: 180
HIS
A: 211
GLN
A: 376
PHE
A: 386
SER
A: 375
N
HIS
A: 363
PHE
A: 207
O
NH
N
ASN
A: 364
O
GLY
A: 372
ASN
A: 371
HIS
A: 367
ILE
A: 368

5

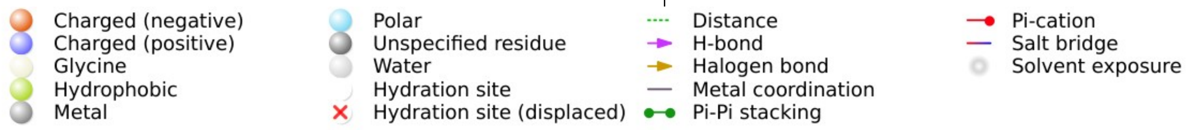
Charged (negative)
Charged (positive)
Glycine
Hydrophobic
Metal
Polar
Unspecified residue
Water
Hydration site
Hydration site (displaced)
Distance
H-bond
Halogen bond
Metal coordination
Pi-Pi stacking
Pi-cation
Salt bridge
Solvent exposure

Figures S1-S10: ^{1}H-NMR and ^{13}C-NMR spectra of synthetized compounds 1-5

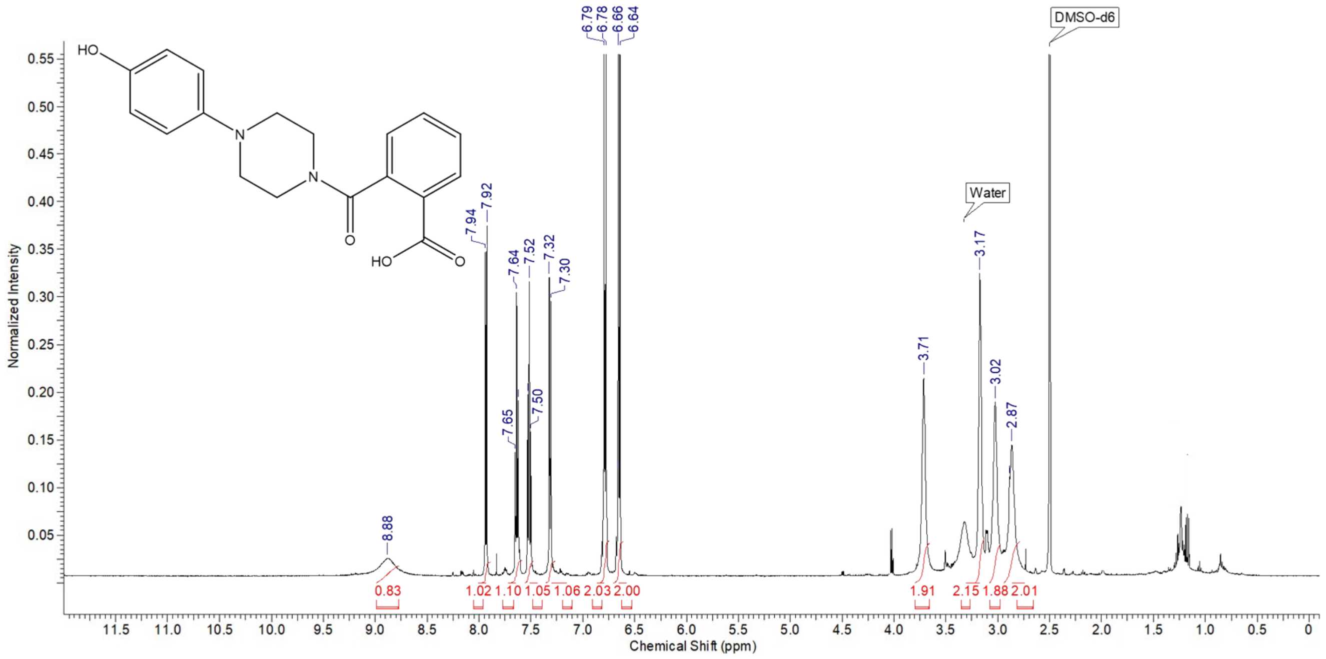

Figure S1. ^{1}H-NMR (DMSO-d_6) spectrum of 2-[4-(4-hydroxyphenyl)piperazine-1-carbonyl]benzoic acid (**1**)

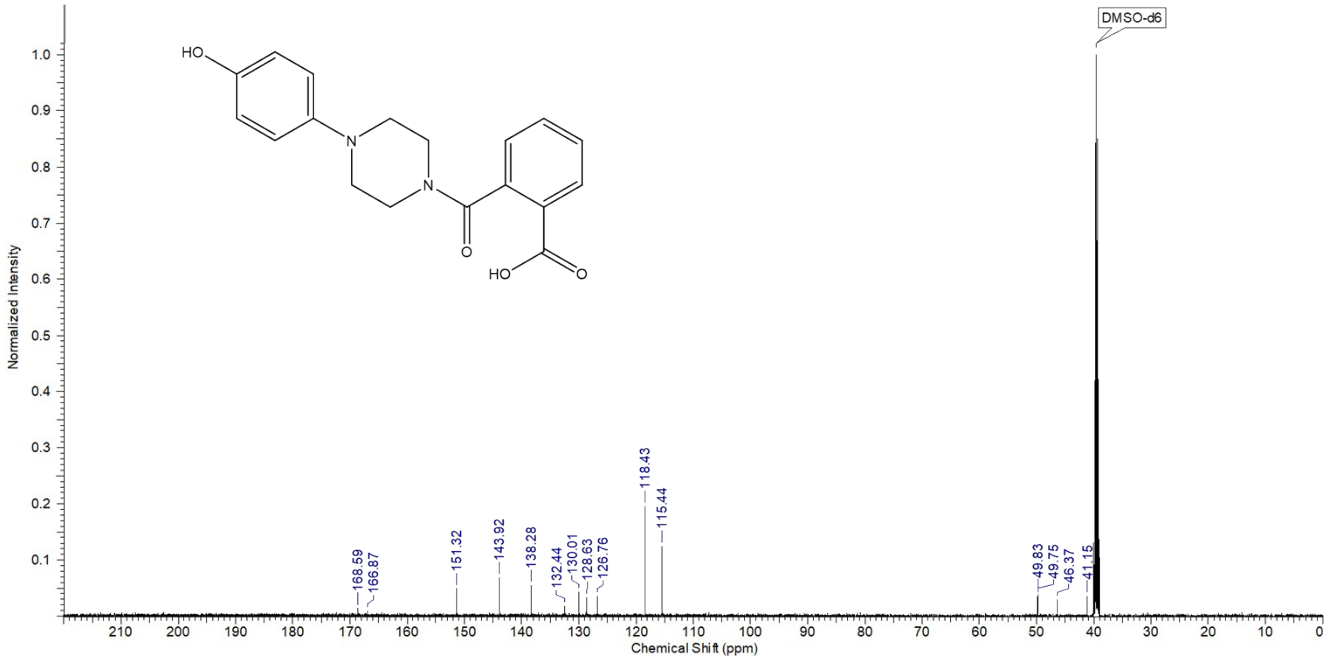

Figure S2. ^{13}C-NMR (DMSO-d_6) spectrum of 2-[4-(4-hydroxyphenyl)piperazine-1-carbonyl]benzoic acid (**1**)

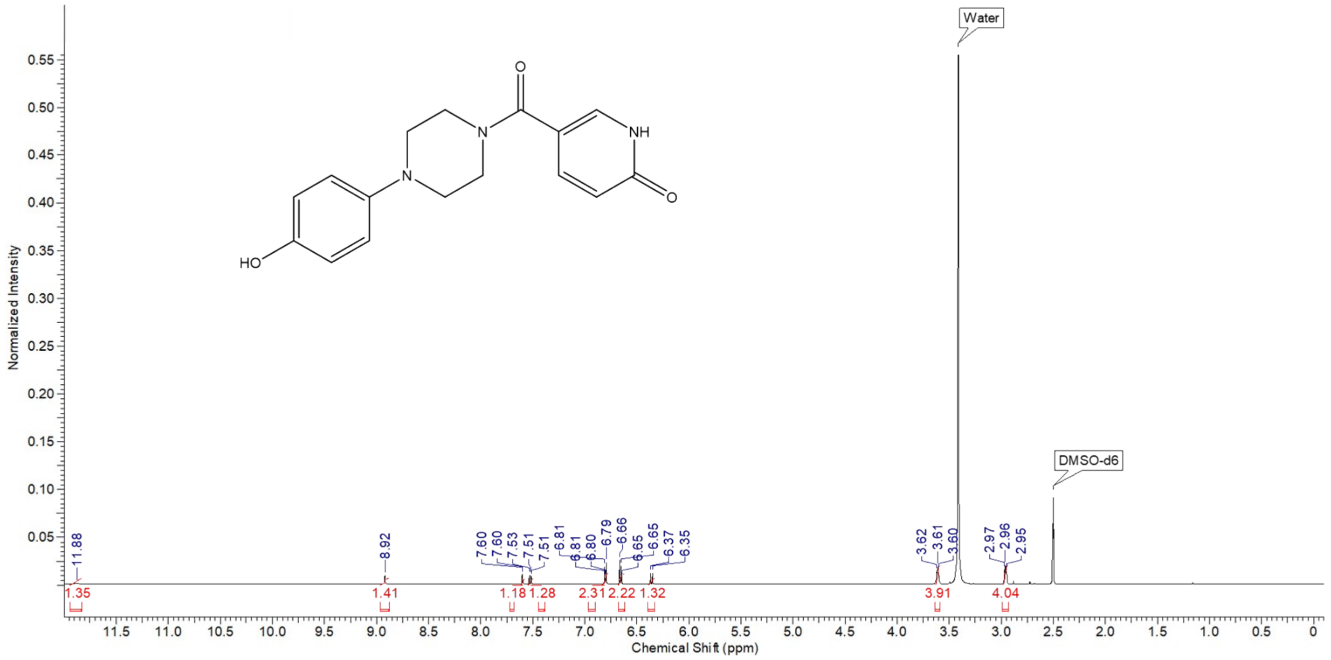

Figure S3. ^{1}H-NMR (DMSO-d_6) spectrum of 5-[4-(4-hydroxyphenyl)piperazine-1-carbonyl]pyridin-2(1H)-one (**2**)

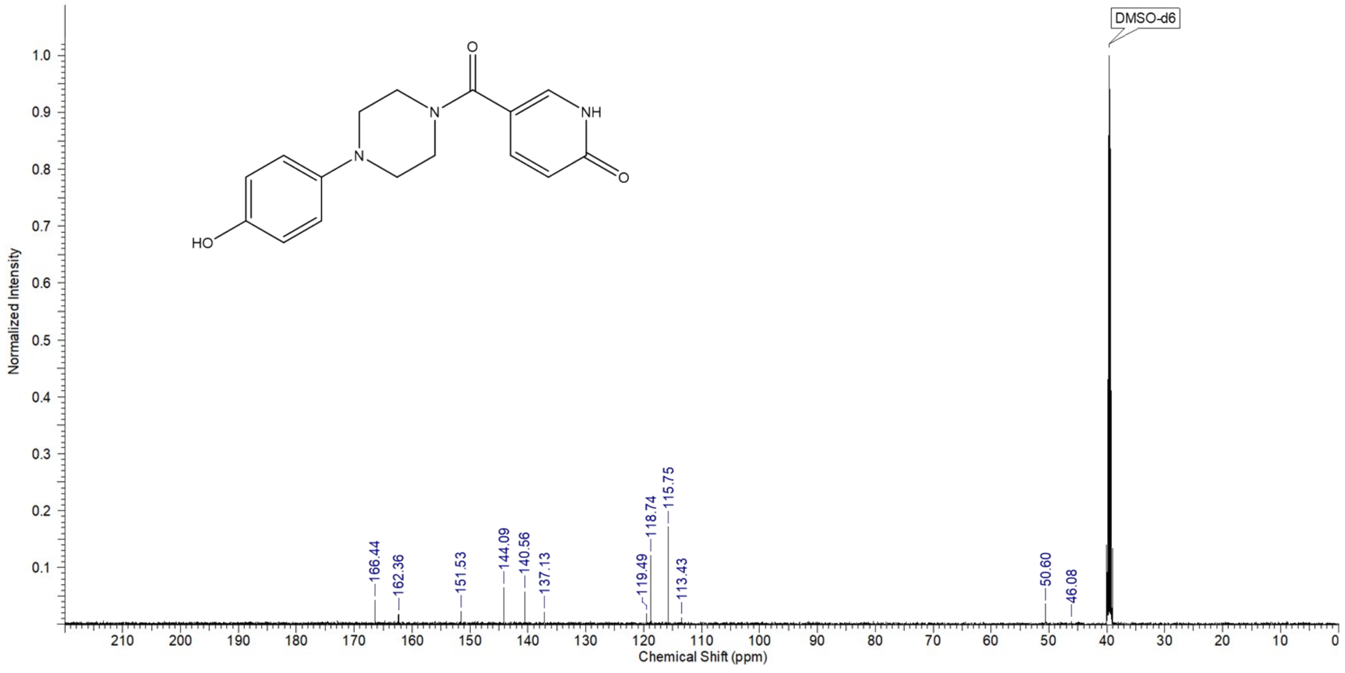

Figure S4. ^{13}C-NMR (DMSO-d_6) spectrum of 5-[4-(4-hydroxyphenyl)piperazine-1-carbonyl]pyridin-2(1H)-one (**2**)

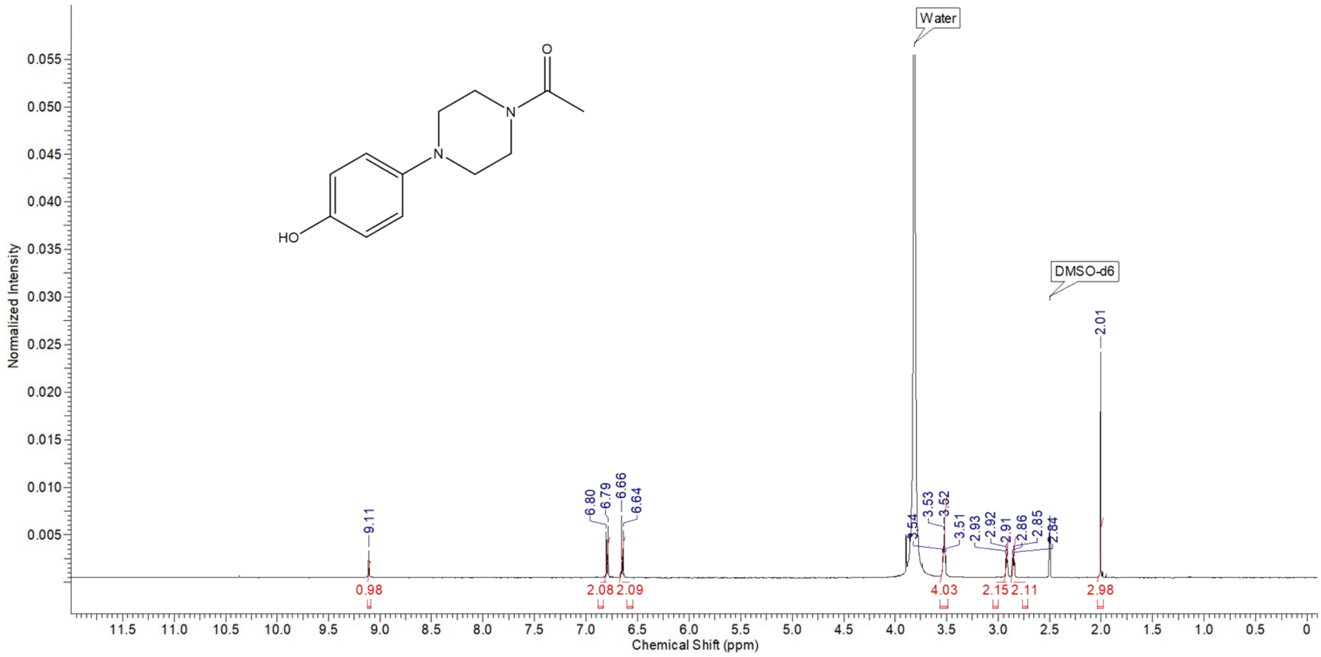

Figure S5. ^{1}H-NMR (DMSO-d_6) spectrum of 1-[4-(4-hydroxyphenyl)piperazin-1-yl]ethan-1-one (**3**)

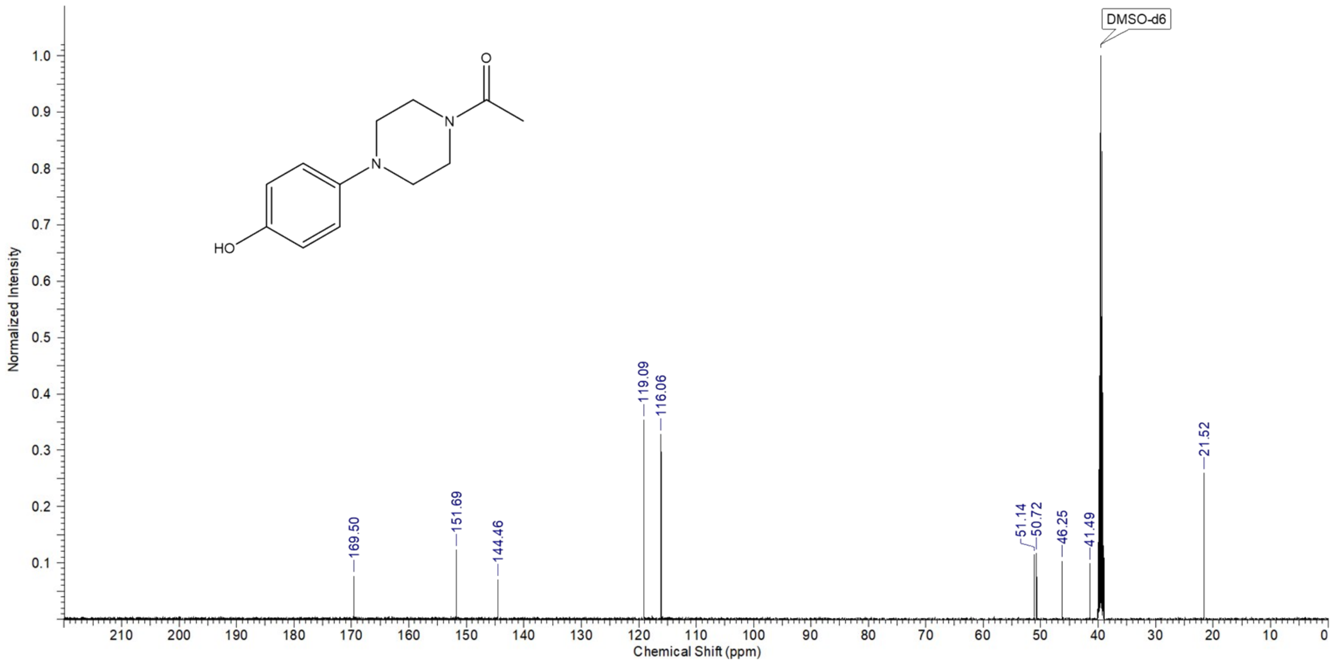

Figure S6. ^{13}C-NMR (DMSO-d_6) spectrum of 1-[4-(4-hydroxyphenyl)piperazin-1-yl]ethan-1-one (**3**)

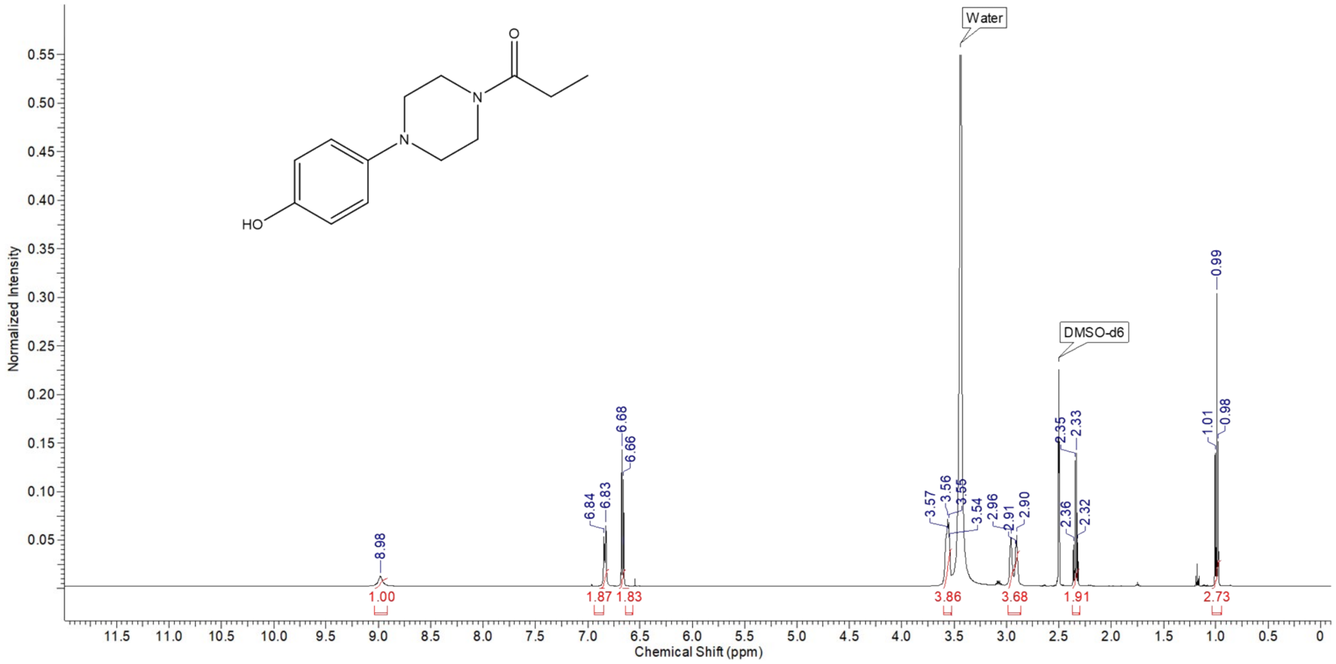

Figure S7. ^{1}H-NMR (500 MHz, DMSO-d_6) spectrum of 1-[4-(4-hydroxyphenyl)piperazin-1-yl]propan-1-one (**4**)

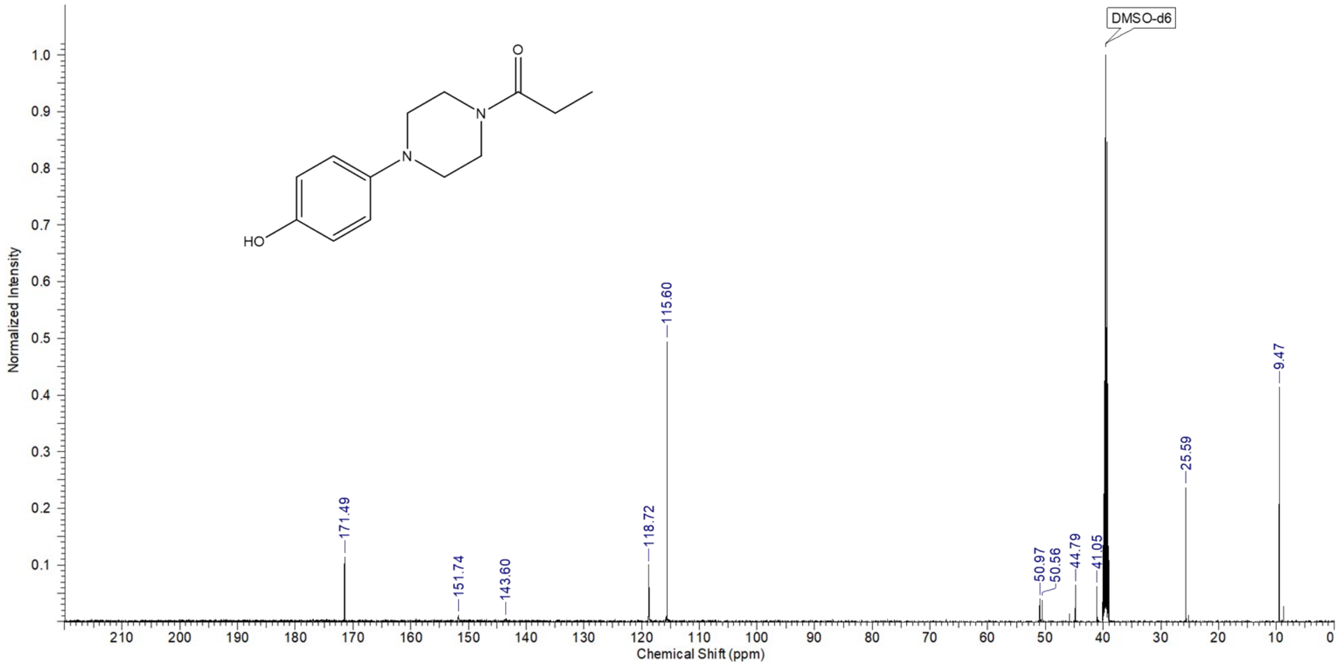

Figure S8. ^{13}C-NMR (126 MHz, DMSO-d_6) spectrum of 1-[4-(4-hydroxyphenyl)piperazin-1-yl]propan-1-one (**4**)

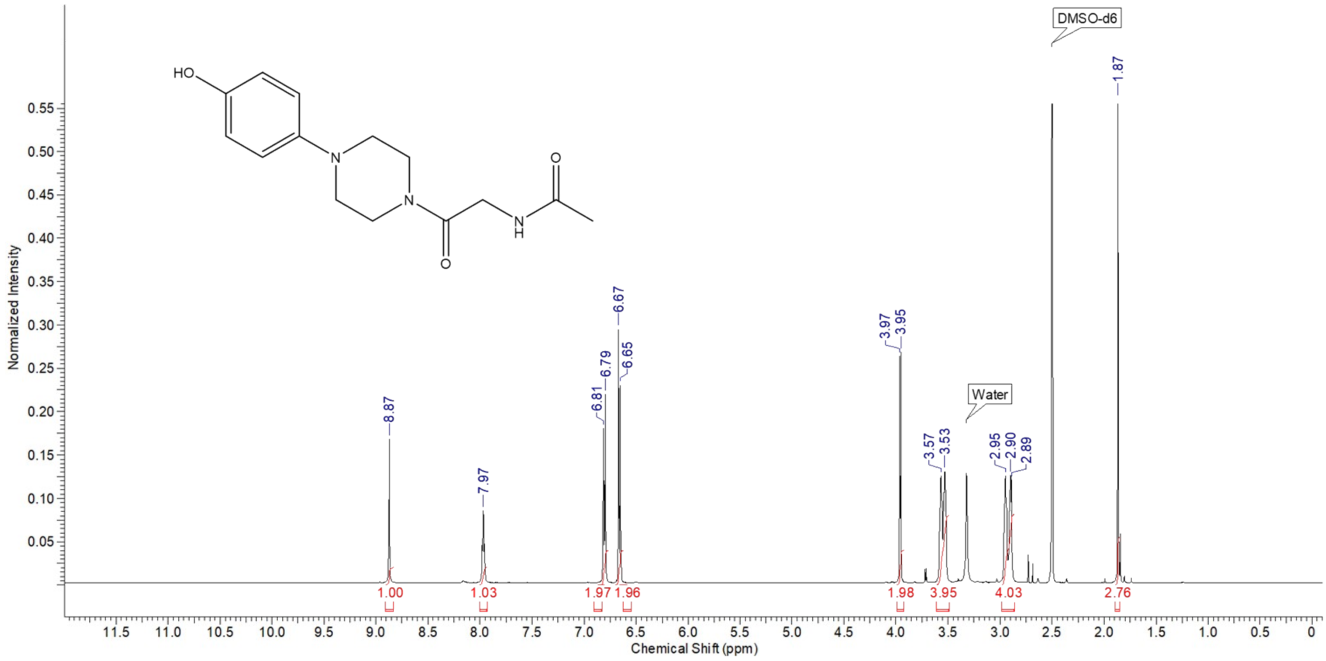

Figure S9. ^{1}H-NMR (DMSO- d_6) spectrum of N-{2-[4-(4-hydroxyphenyl)piperazin-1-yl]-2-oxoethyl}acetamide (**5**)

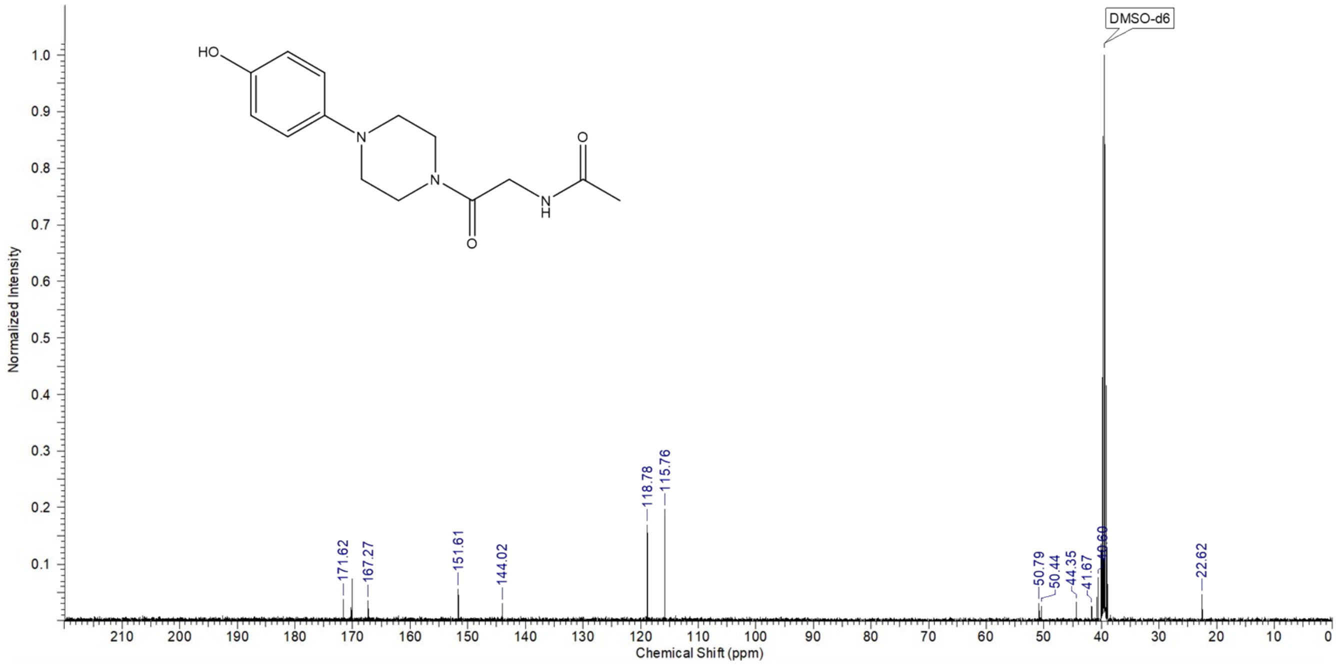

Figure S10. ^{13}C-NMR (DMSO-d_6) spectrum of N-{2-[4-(4-hydroxyphenyl)piperazin-1-yl]-2-oxoethyl}acetamide (**5**)
